# Investigation of bone quality of the first and second sacral segments amongst trauma patients: concerns about iliosacral screw fixation

**DOI:** 10.1007/s10195-015-0354-y

**Published:** 2015-05-28

**Authors:** Dane Salazar, Sean Lannon, Olga Pasternak, Adam Schiff, Laurie Lomasney, Erika Mitchell, Michael Stover

**Affiliations:** The Department of Orthopaedic Surgery and Rehabilitation, Loyola University Health System, 2160 South First Avenue, Maywood, IL 60153, USA; The Department of Radiology, Loyola University Health System, 2160 South First Avenue, Maywood, IL 60153 USA; Department of Orthopaedic Surgery, Feinberg School of Medicine, Northwestern University, 676 N. Saint Clair, Suite 1350, Chicago, IL 60611 USA

**Keywords:** Iliosacral screws, Pelvic fracture fixation, Pelvic ring disruptions, Regional bone density

## Abstract

**Background:**

Iliosacral screw fixation has become a common method for surgical stabilization of acute disruptions of the pelvic ring. Placement of iliosacral screws into the first sacral (S1) body is the preferred method of fixation, but size limitations and sacral dysmorphism may preclude S1 fixation. In these clinical situations, fixation into the second sacral (S2) body has been recommended. The objective of this study was to evaluate the bone quality of the S1 compared to S2 in the described “safe zone” of iliosacral screw fixation in trauma patients.

**Materials and methods:**

The pelvic computed tomography scans of 25 consecutive trauma patients, ages 18–49, at a level 1 trauma center were prospectively analyzed. Hounsfield units, a standardized computed tomography attenuation coefficient, was utilized to measure regional cancellous bone mineral density of the S1 and S2. No change in the clinical protocol or treatment occurred as a consequence of inclusion in this study.

**Results:**

A statically significant difference in bone quality was found when comparing the first and second sacral segment (*p* = 0.0001). Age, gender, or smoking status did not independently affect bone quality.

**Conclusion:**

In relatively young, otherwise healthy trauma patients there is a statistically significant difference in the bone density of the first sacral segment compared to the second sacral segment. This study highlights the need for future biomechanical studies to investigate whether this difference is clinically relevant. Due to the relative osteopenia in the second sacral segment, which may impact the quality of fixation, we feel this technique should be used with caution.

**Level of evidence:**

III

## Introduction

Iliosacral screw fixation has become a common method for surgical stabilization of acute disruptions of the pelvic ring [1–4]. Iliosacral screw placement can be accomplished percutaneously in conjunction with closed reduction or after open reduction; providing stability, minimizing deformity, facilitating mobilization and improving outcomes in patients with posterior pelvic ring injuries [2, 3, 5]. However, loss of fixation, loss of function, neurovascular injury and malunion have all been reported as serious complications following unstable posterior pelvic ring injuries treated using this method [1, 4–8]. Placement of iliosacral screws into the S1 body is the preferred method of fixation, but size limitations and sacral dysmorphism may preclude S1 fixation [4, 9]. In these clinical situations, fixation into the second sacral body (S2) has been recommended [3, 10, 11].

Although safe zones for screw fixation in both normal and dysmorphic second sacral segments have been established, challenges exist in achieving proper fixation into the S2 body because of its smaller size and decreased tolerance of variant screw trajectories [3, 11, 12]. In spite of multiple studies on surgical techniques for optimal placement of fixation, there is little mention of the quality of the surrounding bone in the S2 body. The purpose of this study is to investigate the bone density of the first and second sacral segments using Hounsfield units, a standardized computed tomography attenuation coefficient. We hypothesize that S2 bone density is inferior to that of S1, increasing the chances of screw loosening and fixation failure despite screw placement consistent with accepted methods in the literature.

## Materials and methods

The study was approved by our institutional review board and carried out in the radiology suite of a level 1 trauma center emergency department. Pelvic computed tomography (CT) scans obtained as part of the routine trauma workup of 25 consecutive trauma patients meeting the inclusion criteria were obtained between July 2008 and January 2011. All subjects were between the ages of 18–50 years of age to limit the effects of age-related bone loss or skeletal immaturity. Subjects were excluded from this study for the following reasons: previous documented sacral trauma, presence of a zone 3 sacral fracture, neoplasm of the pelvic girdle, documented history of rheumatoid arthritis, documented history of seronegative arthropathies, documented history of osteoporosis or osteopenia, history of paraplegia, non-ambulatory/wheelchair bound, an inadequate scan technique that would limit density determination, including, but not limited to, motion artifact, streak artifact from internal hardware or external metallic devices, beam-hardening artifact, or photon deprivation in the extremely obese patient, known use of bisphosphonates, steroids and/or hormone medications, or evident malnutrition. The patient’s age, gender and smoking history were recorded from the electronic medical record. No change in protocol or treatment occurred as a consequence of inclusion in this study.

**Fig. 1:**
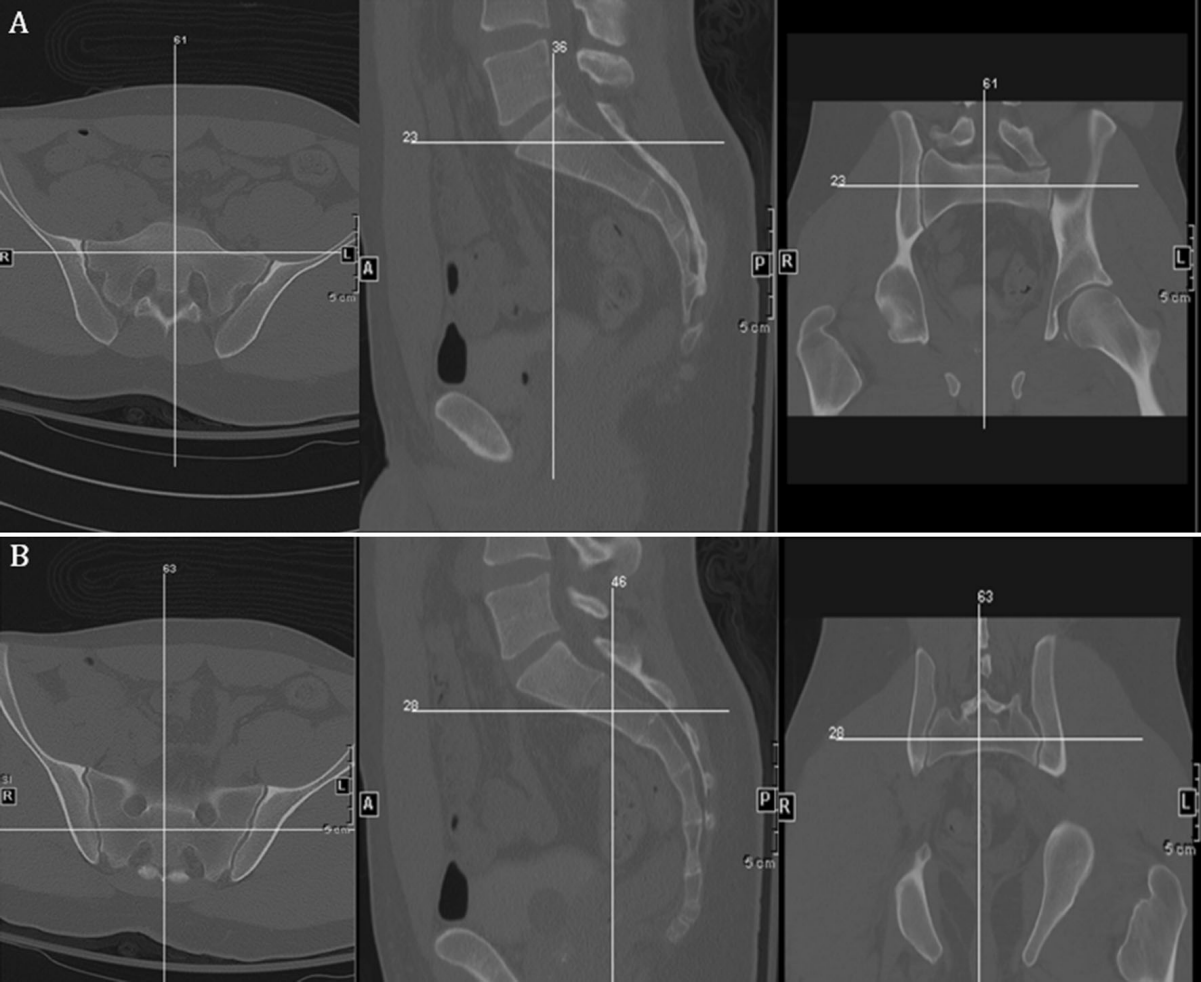
CT images depicting the cross-referencing technique used for localizing the mid body point of S1 (**a**) and S2 (**b**) for measurement in axial, sagittal and coronal reconstructions

**Fig. 2:**
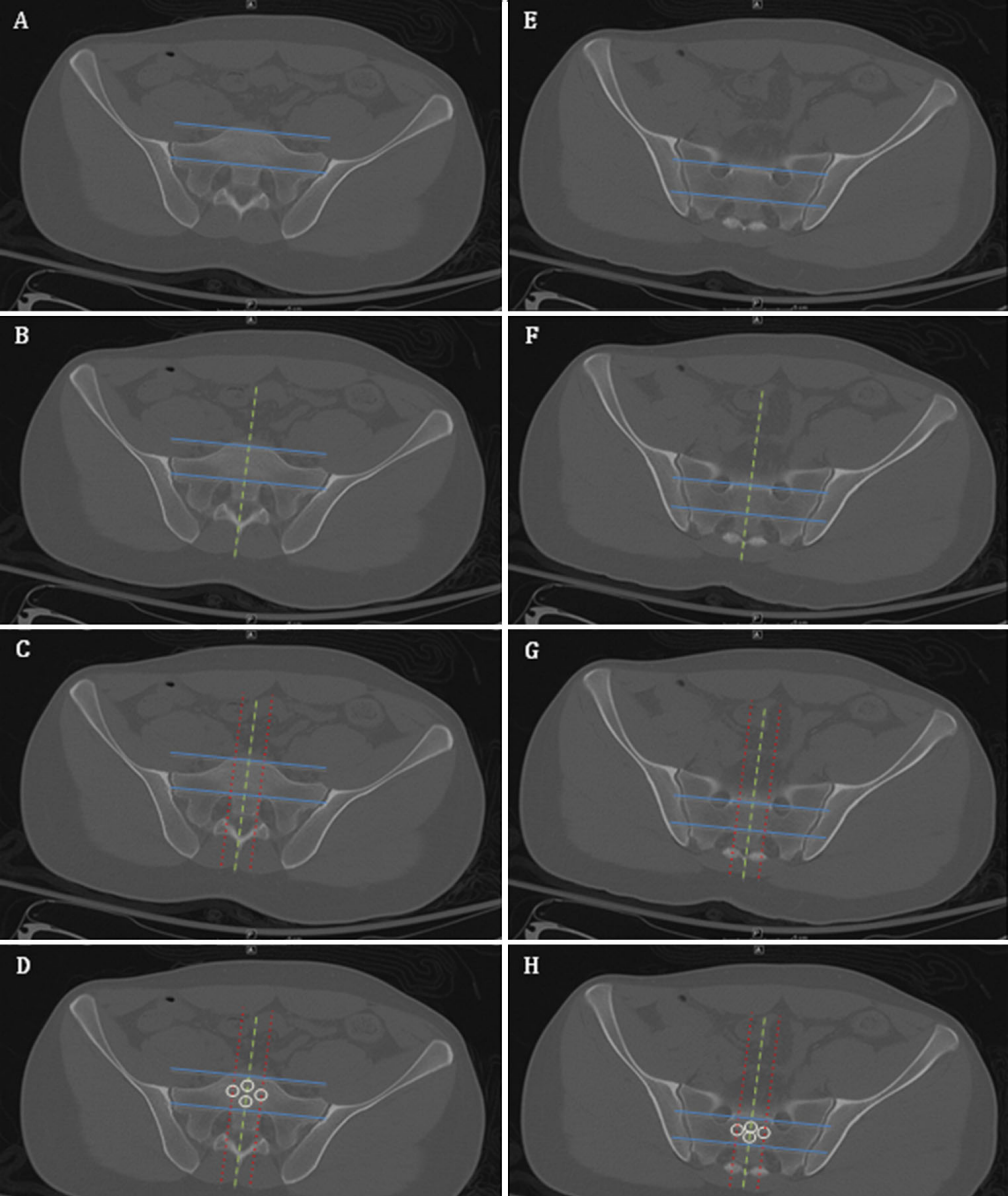
Axial CT sections demonstrating the technique for ROI placement as described in the methods section. **a** *Horizontal reference lines* at S1 (*blue*). **b** *Vertical midline reference line* at S1 (*green dashed*). **c** *Vertical reference line* tangential to the medial border of the sacral foramina at S1 (*red dashed*). **d** Placement of ROIs at 25 and 75 % of the *vertical midline* distance and 50 % of the vertical lateral distance at S1 (*white*). **e**–**h** The same technique at S2 (color figure online)

Images were viewed using the bone algorithm default windows on picture archiving and communication system (PACS) viewing software. Using axial images, the mid-body location of S1 and S2 was determined for each subject and confirmed by cross-referencing position with coronal and sagittal reconstructions (Fig. 1). To standardize measurement while accounting for normal anatomic variation and optimal iliosacral screw trajectory as described in the literature, four standardized circular voxel regions of interests (ROIs) were drawn at determined mid-body S1 and S2 levels of each subject (Fig. 2). These standardized circular ROIs were drawn with areas ranging from 23.2 to 26.2 mm^2^. This range was chosen after pilot testing to maximize the area of trabecular bone tested in line with the potential screw trajectory, while limiting overlap of adjacent ROIs. When placing ROIs, one horizontal reference line was drawn tangential to the most anterior points of both sacral foramina (Fig. 2a, e). One transecting vertical reference line was then drawn from the tip of the spinous process through the midpoint of the anterior cortex of the vertebral body (Fig. 2b, f). ROIs were then drawn with their center corresponding to 25 and 75 % of the distance from the anterior cortex to the horizontal reference line. A vertical reference line was then drawn tangential to the most medial point of the sacral foramina (Fig. 2c, g). An ROI was then drawn with the center of the ROI at 50 % of the distance between the anterior cortex and horizontal reference line drawn previously. This method was then repeated on the adjacent side. Figure 2d and h demonstrates the placement of ROIs. Hounsfield unit (HU) density values for each ROI were then collected and averaged to yield the mean value for each segment.

Prospective power analysis was conducted and revealed that a sample size of 25 patients was necessary to detect a difference in bone density of SI compared to S2 at the 0.05 alpha level with 80 % power. Statistical analysis of the data was performed on the mean values for each segment in the four ROIs examined using paired Student’s *t* tests with statistical significance being set at *p* < 0.05.

## Results

**Fig. 3:**
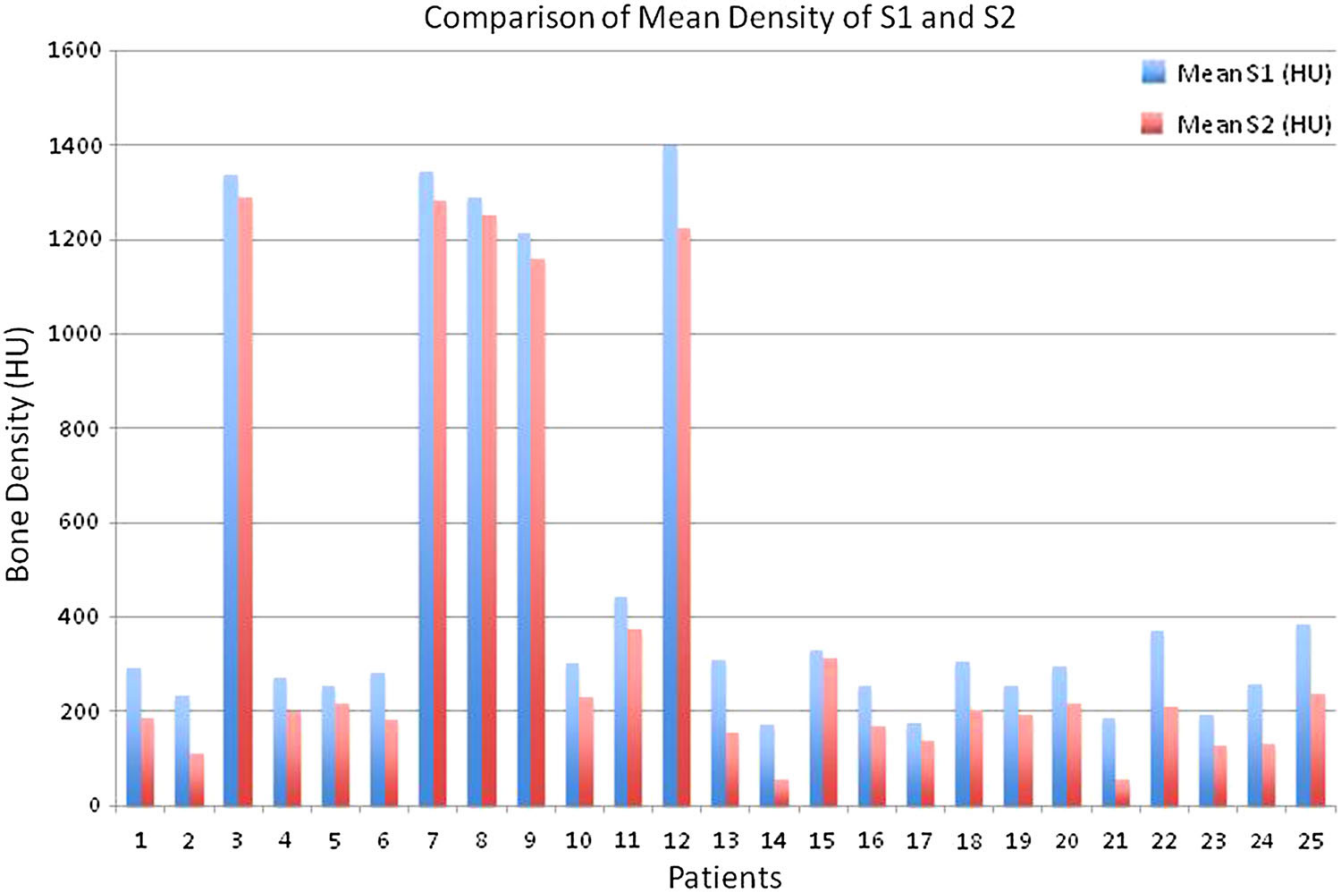
Comparison of mean aggregate bone density measurement of S1 vs. S2 in each subject, as measured in Hounsfield Units (HU)

Twenty-five patients, with a mean age of 35.2 years, were studied (ages 18–49 years). Thirteen patients had a positive smoking history. Nineteen patients were male and 6 female. The difference between the average Hounsfield unit (HU) of the first and second sacral segment was 89.9 (*p* = 0.0001). Comparisons of the mean bone density of the first and second sacral segments are presented in Fig. 3.

The 13 patients with a positive smoking history had a mean HU of 93.7 compared to the mean HU value of non-smokers, 85.8 (*p* = 0.66). The average HU difference when comparing males, 87.2, versus females, 98.6, was 11.4 (*p* = 0.58). Age had no significant effect on HU difference (*p* = 0.53).

The values of bone mineral density in Hounsfield units for each of the tested points are detailed in Table 1 for each of the patients. All four points were found to have a statistical difference between S1 and S2 (anterior *p* = 0.0011, posterior *p* < 0.0001, right *p* < 0.0001, left *p* < 0.0001). The percentage difference of mean density measured with Hounsfield units between S1 and S2 is presented in Table 2.

**Table 1:** Values of bone mineral density in Hounsfield units of analogous ROI locations at S1 and S2 with differences

Patient no.	S1 anterior	S2 anterior	Difference	S1 posterior	S2 posterior	Difference 2	S1 right	S2 right	Difference 3	S1 left	S2 left	Difference 4
1	295.9	226.9	69	261.5	205	56.5	321.2	158.9	162.3	288.1	156.3	131.8
2	240.9	140.9	100	170.3	98.9	71.4	270.2	106.1	164.1	259.7	92.3	167.4
3	1313	1272.4	40.6	1358.7	1298.6	60.1	1369	1294.5	74.5	1310	1288.7	21.3
4	280.9	236.2	44.7	211.8	200	11.8	296.7	186.8	109.9	294.8	174.3	120.5
5	229.5	239.3	−9.8	263	164.9	98.1	263	218.1	44.9	256.8	244.3	12.5
6	230.1	236.3	−6.2	256.7	134.9	121.8	313.5	177.5	136	323.8	180.7	143.1
7	1341.3	1279.4	61.9	1348.8	1262.9	85.9	1335.9	1306.1	29.8	1355.6	1277.3	78.3
8	1342.1	1279.2	62.9	1292.7	1265.5	27.2	1263.3	1237.2	26.1	1258.9	1222.2	36.7
9	1292.3	1237.3	55	1263.8	1146.6	117.2	1148.1	1103.6	44.5	1154.5	1142.2	12.3
10	306.7	268.6	38.1	331.4	200.1	131.3	266.5	222.4	44.1	307	224.7	82.3
11	361.4	373.9	−12.5	547.3	391.2	156.1	447.3	344.5	102.8	411.9	393.5	18.4
12	1392.7	1230.8	161.9	1389.2	1234.3	154.9	1398.8	1191.3	207.5	1417.7	1240.4	177.3
13	293.4	233.4	60	393.3	80.3	313	283.4	160.8	122.6	267.9	153.4	114.5
14	139.5	97	42.5	138.5	41.9	96.6	190	38.7	151.3	216.6	43	173.6
15	287.1	408.7	−121.6	341.9	290.6	51.3	379	256.7	122.3	309.1	296.6	12.5
16	247.8	223	24.8	212.3	151.7	60.6	305.2	136	169.2	254.1	163.2	90.9
17	212.2	167.7	44.5	188.2	106.2	82	150.2	152.6	−2.4	146.1	124.3	21.8
18	248.7	277.9	−29.2	261.7	158	103.7	388.4	197.2	191.2	328.5	173.2	155.3
19	223.4	196.3	27.1	248.9	215.2	33.7	268.2	139.7	128.5	275.3	220.4	54.9
20	271.7	279.3	−7.6	292.4	189.4	103	305.2	217.8	87.4	317.7	176.8	140.9
21	207.9	77.9	130	235	58	177	128.1	39.3	88.8	167.1	44.5	122.6
22	407.5	207.7	199.8	401.9	202.4	199.5	311.9	191.3	120.6	365.2	237.7	127.5
23	155.5	129.4	26.1	162.1	166.2	−4.1	254.9	97.6	157.3	203.3	119.1	84.2
24	367.5	181.4	186.1	172.5	66.4	106.1	232.7	143.9	88.8	258.8	137.9	120.9
25	422.2	306.8	115.4	370.9	223.9	147	384.3	196.2	188.1	367.5	224	143.5
			52.14 (*p* = 0.0011)			102.47 (*p* < 0.0001)			110.41 (*p* < 0.0001)			94.6 (*p* < 0.0001)

All values listed are in Hounsfield units

**Table 2:** Values of bone mineral density in Hounsfield units of analogous ROI locations at S1 and S2 with differences

Patient no.	Difference anterior ROI	% Difference S2 vs. S1 anterior ROI	Difference posterior ROI	% Difference S2 vs. S1 anterior ROI	Difference right ROI	% Difference S2 vs. S1 anterior ROI	Difference left ROI	% Difference S2 vs. S1 anterior ROI
1	69	77	57	78	162	49	132	54
2	100	58	71	58	164	39	167	36
3	41	97	60	96	75	95	21	98
4	45	84	12	94	110	63	121	59
5	−10	104	98	63	45	83	13	95
6	−6	103	122	53	136	57	143	56
7	62	95	86	94	30	98	78	94
8	63	95	27	98	26	98	37	97
9	55	96	117	91	45	96	12	99
10	38	88	131	60	44	83	82	73
11	−13	103	156	71	103	77	18	96
12	162	88	155	89	208	85	177	87
13	60	80	313	20	123	57	115	57
14	43	70	97	30	151	20	174	20
15	−122	142	51	85	122	68	13	96
16	25	90	61	71	169	45	91	64
17	45	79	82	56	−2	102	22	85
18	−29	112	104	60	191	51	155	53
19	27	88	34	86	129	52	55	80
20	−8	103	103	65	87	71	141	56
21	130	37	177	25	89	31	123	27
22	200	51	200	50	121	61	128	65
23	26	83	−4	103	157	38	84	59
24	186	49	106	38	89	62	121	53
25	115	73	147	60	188	51	144	61
Mean difference (*p* value)	52 (*p* = 0.0011)		102 (*p* < 0.0001)		110 (*p* < 0.0001)		95 (*p* < 0.0001)	
Percent difference of S2 compared to S1		86		68		65		69
					Average global density of S2 compared to S1			71.9

## Discussion

Iliosacral screw fixation has emerged as the treatment of choice for unstable injuries involving the posterior pelvic ring. However, the posterior pelvic anatomy is complex and variable, and thus placement of fixation can be technically challenging. A 44 % incidence of sacral dysmorphism has been reported; therefore, a thorough understanding of the typical as well as atypical individual anatomy is critical for reliably placing safe iliosacral screws [3, 11]. In dysmorphic sacra, the first sacral safe zone was 36 % smaller compared to the normal counterparts, and with more oblique orientation from caudal to cranial and posterior to anterior [11]. In the second segment safe zone, the cross-sectional area was more than twice as large in the dysmorphic sacra compared to normal [11]. Additionally, it was found that a transverse screw could be safely placed at the S2 level in 95 % of dysmorphic sacra but only in 50 % of normal sacra [11].

The optimal fixation construct remains unclear; however, injuries with multiplanar instability have increased the rates of fixation failure [13]. Biomechanical studies have suggested improved stability using two points of posterior fixation for the treatment of unstable pelvic ring injuries [14, 15]. Therefore, the placement of two fixation screws has been recommended to aid with stability. Several clinical scenarios necessitate the placement of fixation into the second sacral segment.

Multiple cadaveric and in vivo studies have investigated proving the efficacy and safety of S2 screw fixation using both fluoroscopic and computer tomography-based multiplanar guidance systems to identify reliable and reproducible landmarks to establish a safe corridor [10, 12, 16–19]. Several case series have established the placement of fixation into the second sacral segment as a dependable alternative or adjunct fixation method to the more common first sacral segment [3, 4, 13].

However there is a paucity of data examining the quality of bone of the second sacral segment compared to the first sacral segment. In one clinical series with 62 patients treated with closed reduction and placement of percutaneous iliosacral screws for unstable pelvic ring injuries, 2 patients were managed with 2 S1 screws, 3 with 2 screws in S2, 56 with 1 S1 and another in S2, and 1 patient with 2 screws in S1 and a 3rd in S2. Fixation failure occurred in 4 of 62 patients. Retrospectively, five patients were identified as being osteopenic, with two of these five patients having early fixation failure. This led the authors to conclude that S2 screws should be used with caution in patients with suspected pelvic and sacral osteopenia/osteoporosis [13]. Additionally, in a series of 49 patients all treated with S2 screws, 2 had postoperative loss of reduction requiring revision surgery, both with radiographic evidence of osteopenia. This led to the recommendation of finding alternative fixation methods in those patients with osteopenia and in patients with questionable intraoperative screw purchase during placement [3]. To our knowledge, our study is the first to specifically compare the bone densities of the first two sacral segments.

Multiple modalities of measuring bone density have been described and validated, including dual X-ray absorptiometry (DEXA), plain radiographs and quantitative computed tomography [20]. More recent studies have demonstrated that computed tomography examinations utilizing automatic exposure control are able to accurately measure regional cancellous bone mineral density [21]. In our study we utilized Hounsfield units, a standardized computed tomography attenuation coefficient, which has been shown to correlate with both the DEXA and compressive strengths of osseous models. We hypothesized that S2 bone density is inferior to that of S1, increasing the chances of screw loosening and fixation failure despite screw placement consistent with accepted methods in the literature.

We prospectively assessed the pelvic computed tomography scans of 25 consecutive trauma patients evaluated in the Emergency Department of a level 1 trauma center. We found a statistically significant difference in the bone density at all four points and the aggregate of S1 compared to S2. Smoking history, gender and age were not found to be independent factors in contributing to this difference.

One of the limitations of our study is that Hounsfield units on computed tomography were used as a surrogate measurements of “bone density” or “bone quality.” This non-invasive method is well described in the literature [21] and has previously been utilized as a tool to draw conclusions about bone mineral density; however, it should be noted that it is a quantitative and not a qualitative measurement. To directly calculate bone quality and thus truly investigate the local trabecular microarchitecture of bone, would require a bone biopsy.

The optimal fixation for posterior pelvic ring injuries remains unclear. Our study demonstrates that in relatively young, otherwise healthy trauma patients there is a statistically significant difference in the bone density of the first sacral segment compared to the second sacral segment. This study highlights the need for future biomechanical studies to investigate whether this difference has a clinically relevant effect on the quality of fixation. Previous studies have highlighted clinical scenarios in which fixation in the second sacral segment is warranted and have proposed that this technique is safe and effective. However, given our findings of relative osteopenia in the second sacral segment, which may impact the quality of fixation, we feel this technique should be used with caution.

## References

[1] Matta JM, Saucedo T (1989) Internal fixation of pelvic ring fractures. Clin Orthop Relat Res 83–97 2706863

[2] Matta JM, Tornetta P 3rd (1996) Internal fixation of unstable pelvic ring injuries. Clin Orthop Relat Res 129–140 10.1097/00003086-199608000-00016 8769444

[3] Moed BR, Geer BL (2006). S2 iliosacral screw fixation for disruptions of the posterior pelvic ring: a report of 49 cases. J Orthop Trauma.

[4] Routt ML Jr, Simonian PT (1996) Closed reduction and percutaneous skeletal fixation of sacral fractures. Clin Orthop Relat Res 121–128 10.1097/00003086-199608000-00015 8769443

[5] Shuler TE, Boone DC, Gruen GS (1995). Percutaneous iliosacral screw fixation: early treatment for unstable posterior pelvic ring disruptions. J Trauma.

[6] Keating JF, Werier J, Blachut P (1999). Early fixation of the vertically unstable pelvis: the role of iliosacral screw fixation of the posterior lesion. J Orthop Trauma.

[7] Tonetti J, Cloppet O, Clerc M (2000). Implantation of iliosacral screws. Simulation of optimal placement by 3-dimensional X-ray computed tomography. Revue de chirurgie orthopedique et reparatrice de l’appareil moteur.

[8] Cole JD, Blum DA, Ansel LJ (1996) Outcome after fixation of unstable posterior pelvic ring injuries. Clin Orthop Relat Res 160–179 10.1097/00003086-199608000-00020 8769448

[9] Conflitti JM, Graves ML, Chip Routt ML Jr (2010) Radiographic quantification and analysis of dysmorphic upper sacral osseous anatomy and associated iliosacral screw insertions. J Orthop Trauma 24:630–636 10.1097/BOT.0b013e3181dc50cd 20871251

[10] Carlson DA, Scheid DK, Maar DC (2000). Safe placement of S1 and S2 iliosacral screws: the “vestibule” concept. J Orthop Trauma.

[11] Gardner MJ, Morshed S, Nork SE (2010). Quantification of the upper and second sacral segment safe zones in normal and dysmorphic sacra. J Orthop Trauma.

[12] Gautier E, Bachler R, Heini PF et al (2001) Accuracy of computer-guided screw fixation of the sacroiliac joint. Clin Orthop Relat Res 310–317 10.1097/00003086-200112000-00036 11764364

[13] Griffin DR, Starr AJ, Reinert CM et al (2006) Vertically unstable pelvic fractures fixed with percutaneous iliosacral screws: does posterior injury pattern predict fixation failure? J Orthop Trauma 20:S30–S36 **(discussion S36****)** 16385205

[14] van Zwienen CM, van den Bosch EW, Snijders CJ (2004). Biomechanical comparison of sacroiliac screw techniques for unstable pelvic ring fractures. J Orthop Trauma.

[15] Yinger K, Scalise J, Olson SA (2003). Biomechanical comparison of posterior pelvic ring fixation. J Orthop Trauma.

[16] Arman C, Naderi S, Kiray A (2009). The human sacrum and safe approaches for screw placement. J Clin Neurosci.

[17] Hinsche AF, Giannoudis PV, Smith RM (2002) Fluoroscopy-based multiplanar image guidance for insertion of sacroiliac screws. Clin Orthop Relat Res 135–144 10.1097/00003086-200202000-00014 11937873

[18] Nottmeier EW, Pirris SM, Balseiro S (2010). Three-dimensional image-guided placement of S2 alar screws to adjunct or salvage lumbosacral fixation. Spine J.

[19] Ziran BH, Smith WR, Towers J (2003). Iliosacral screw fixation of the posterior pelvic ring using local anaesthesia and computerised tomography. J Bone Joint Surg Br.

[20] Grampp S, Genant HK, Mathur A (1997). Comparisons of noninvasive bone mineral measurements in assessing age-related loss, fracture discrimination, and diagnostic classification. J Bone Miner Res.

[21] Schreiber JJ, Anderson PA, Rosas HG (2011). Hounsfield units for assessing bone mineral density and strength: a tool for osteoporosis management. J Bone Joint Surg Am.

